# Efficacy of indigo naturalis nanofibrous patches in the treatment of chronic plaque psoriasis: a 4-week prospective, randomized, semi-compartmental paired, positive drug-controlled trial

**DOI:** 10.3389/fphar.2025.1595019

**Published:** 2025-06-06

**Authors:** Yuechun Zhao, Jiaqi Li, Ningxin Zhang, Mingyue Liu, Pengyu Wang, Ning Li, Ruodan Xu, Ping Song

**Affiliations:** ^1^ Department of Dermatology, Xiyuan Hospital of China Academy of Chinese Medical Sciences, Beijing, China; ^2^ Department of Biomedical Engineering and Technology, Institute of Basic Theory for Chinese Medicine, China Academy of Chinese Medical Sciences, Beijing, China

**Keywords:** psoriasis, indigo naturalis, clinical trial, nanofibrous patch, calcipotriol

## Abstract

**Background:**

Indigo Naturalis (IN) has been traditionally used for psoriasis treatment. We developed an IN-poly (ε -caprolactone, PCL)/poly (ethylene oxide, PEO) nanofibrous patch using electrospinning technology. Preliminary studies demonstrated its potential to reduces keratinocyte proliferation, inflammation, and neovascularization, addressing limitations of conventional IN formulations such as poor absorption and skin staining.

**Aims:**

To evaluate the safety and efficacy of the IN-PCL/PEO patch for chronic plaque psoriasis.

**Methods:**

The clinical trial included 36 patients diagnosed with chronic plaque psoriasis. Two symmetrical psoriatic lesions from each eligible patient were selected by dermatologists and randomly assigned to receive either calcipotriol ointment (control group) or IN-PCL/PEO nanofibrous patch (test group) for 4 weeks. Primary outcomes included Psoriasis Area and Severity Index (PASI), while secondary outcomes assessed Patient Global Assessment (PGA), skin irritation, and patient satisfaction. Safety was monitored via blood, urine, and liver/kidney function tests.

**Results:**

30 subjects completed the treatment trial with both IN and CA. In the test group (n = 30), PASI scores decreased from 6.30 ± 2.23 to 2.13 ± 1.57 (*p* < 0.001). Within this group, 90.0% (27/30) achieved PASI 50% and 43.3% (13/30) achieved PASI 75. No skin irritation cases were reported (0.0%). While PASI scores decreased from 6.23 ± 2.27 to 2.23 ± 1.50 (*p* < 0.001) in the control group, 86.7% (26/30) achieved PASI 50% and 33.3% (10/30) achieved PASI 75, with 6.7% (2/30) experiencing irritation. All safety parameters remained within standard reference ranges.

**Conclusion:**

The IN-PCL/PEO patch demonstrated efficacy comparable to calcipotriol ointment, with improved safety and tolerability, suggesting its potential as an alternative psoriasis treatment.

## Graphical Abstract

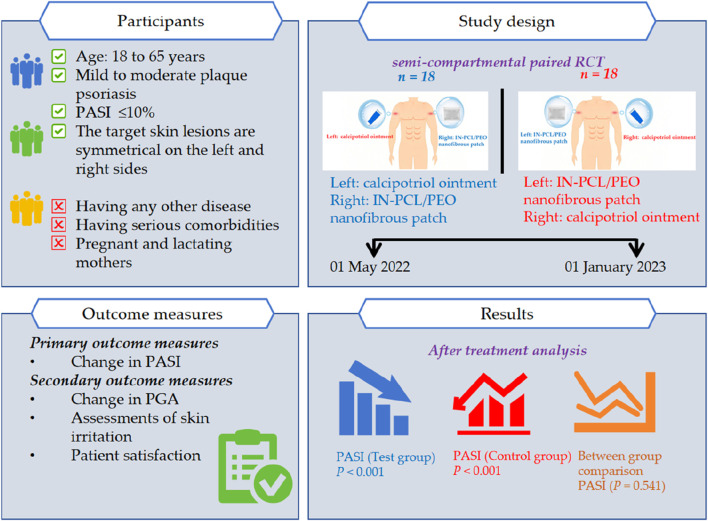

## 1 Introduction

Psoriasis is a prevalent chronic inflammatory dermatological condition affecting an estimated 29.5 million adults globally, with prevalence rates ranging from 0.05% to 1.88% worldwide (Parisi et al., 2020). The disease is characterized by well-demarcated, scaly (silvery-white), inflammatory, and pruritic plaques, which predominantly manifest on the extensor surfaces of the elbows and knees, although they may also appear on the scalp and back. This condition substantially impacts the patient’s quality of life, contributing to physical and psychological distress (Arampatzis et al., 2021). The pathogenesis of psoriasis is driven by a complex interplay of genetic, immunological, and environmental factors, primarily triggered by the activation of T-cell subsets, including CD8^+^ T-cells, Th1 cells, autoreactive T-cells, as well as Th17 and Th22 cells, which culminates in localized inflammation and keratinocyte hyperproliferation (Skrek et al., 2023; Potestio et al., 2024; Griffiths and Barker, 2007; Kielbowski et al., 2024). The therapeutic strategy for managing psoriasis is typically guided by the severity of the disease and the extent of the affected in the body’s surface area (Timis et al., 2021). Conventional modalities encompass systemic, topical, physiotherapy, and biological therapies (Chang et al., 2019; Nowowiejska et al., 2021). Although these treatments effectively manage inflammation and alleviate symptoms, notable side effects remain a concern. For instance, Systemic therapies like methotrexate are associated with hepatotoxicity, nephrotoxicity, and potential damage to the endocrine and reproductive systems (Caldarola et al., 2023; Campbell and Laws, 2024; Messana et al., 1990). Additionally, biologics and oral small molecule inhibitors, including Tofacitinib, despite their efficacy, face limitations in accessibility due to high costs and contraindications, and their long-term effects remain an open question (Zhang L. et al., 2022; Hwang et al., 2023; Chiricozzi et al., 2015). In contrast, topical treatments have emerged as the preferred option for mild to moderate psoriasis due to their lower side effects, convenience, and cost-effectiveness. Nevertheless, topical medications, including calcipotriene ointment, tazarotene, and corticosteroids, may associated with adverse effects such as skin irritation, photosensitivity reactions, and long-term consequences like skin atrophy and telangiectasia (Raharja et al., 2021; Greb et al., 2016). Consequently, the development of novel alternative therapies is urgently required.

Indigo naturalis (IN) is an effective traditional Chinese medicine (TCM) herb utilized in clinical practice to treat psoriasis (Zhang Q. et al., 2022; Wang et al., 2024). As early as in the Kaibao Materia Medica (Song Dynasty, 973 AD) and Compendium of Materia Medica (Ming Dynasty, 1590 AD), IN was documented for its multiple therapeutic properties, including antipyretic, detoxifying, cooling blood, hemostatic, liver-clearing, and fire-reducing effects, and was widely used in the treatment of skin diseases. Modern pharmacological studies have revealed that IN exerts its therapeutic effects on psoriasis by regulating cell apoptosis, proliferation, and differentiation, inhibiting inflammatory responses, and modulating angiogenesis (Qi-Yue et al., 2020). In the early stages of our research, we achieved favorable clinical outcomes with IN Compound ointment in treating psoriasis in a single-center study (Wang et al., 2023). Nevertheless, as a semisolid ointment, IN Compound ointment presents challenges in dose control, insufficient transdermal absorption, a sticky and greasy texture, and dark color, which often leaves undesirable residues on the skin and clothing, reducing patient adherence and consequently affecting treatment efficacy (Xu et al., 2024).

Fortunately, with the advancement of nanotechnology in recent years, transdermal drug delivery systems have significantly improved. Nanofiber membranes prepared by electrospinning, with their high surface area and porosity, facilitate enhanced transdermal drug absorption (Han et al., 2024; Su et al., 2024; Zhou et al., 2024). Leveraging these advantages, we previously developed a functionalized IN-loaded PCL/PEO amphiphilic nanofiber membrane (IN-PCL/PEO), and through *in vitro* cellular experiments and imiquimod-induced psoriasis mouse models, we validated its safety and efficacy through *in vitro* cellular experiments and imiquimod (IMQ)-induced psoriasis mouse models (Guo et al., 2022; Li et al., 2021). However, there is still limited clinical evidence regarding the effectiveness of IN-PCL/PEO membranes in psoriasis patients. Therefore, we conducted a randomized, semi-compartmental paired, positive drug-controlled clinical trial to evaluate the clinical efficacy and safety of IN-PCL/PEO nanofiber patches in treating psoriasis.

## 2 Materials and methods

### 2.1 Study design

This single-center, randomized, open-label, positive drug-controlled prospective clinical trial enrolled patients with vulgaris psoriasis from May 2022 to January 2023 at the dermatology outpatient clinic in Guang’anmen Hospital, China Academy of Chinese Medical Sciences. To control individual differences and reduce confounding factors, symmetrical lesions on both sides of the participants’ bodies were selected. Using sealed-envelope randomization, participants were allocated in a 1:1 ratio to two groups: In Group 1 (n = 18), the left target lesions were treated with IN-PCL/PEO nanofibrous patches (test group) while the right lesions were treated with calcipotriol ointment (control group); Group 2 (n = 18) received the reverse treatment regimen. This self-controlled design ultimately yielded 36 paired lesions for both test and control groups. All the participants underwent five scheduled visits at 0, 1, 2, 3, and 4 weeks. The study was approved by the Ethics Committee of Guang’anmen Hospital, China Academy of Chinese Medical Sciences (Approval No. 2022–214-KY). It was conducted following the principles outlined in the Helsinki Declaration for Humans while adhering to the reporting standards for randomized controlled trials. All study participants provided written informed consent to participate in the trial. The study adhered to CONSORT guidelines for randomized trials.

### 2.2 Participants

The diagnostic criteria for vulgaris psoriasis are based on the criteria set by the EuroGuiDerm Guideline in 2021 (Nast et al., 2021). The inclusion criteria were: (1) Patients aged 18–65 years, either male or female, with a clinical diagnosis of mild to moderate plaque psoriasis and a Psoriasis Area Severity Index (PASI) score of less than 10%. (2) Fulfill vulgaris psoriasis diagnostic criteria: the presence of well-defined, dark red, or erythematous plaques, often covered with characteristic white or silvery-white scales. These plaques may exhibit a waxing and waning course, and can be associated with features such as a thin scale layer and pinpoint hemorrhages (Auspitz’s sign) (Vázquez López et al., 2014; Dias et al., 2023). (3) Willingness to provide written informed consent.

The exclusion criteria for this study were as follows: patients with known or suspected hypersensitivity to the investigational drug or its components; those currently using concomitant medications or treatments that are contraindicated or for which discontinuation is not feasible during the study period; individuals with severe comorbidities, including cardiovascular, neurological, hepatic, renal, hematologic disorders, or malignancies; women who are pregnant, breastfeeding, or planning to conceive during the study period; patients with a history of alcohol or drug abuse; individuals with psychiatric or neurological disorders that could impair cooperation; participants who had enrolled in other clinical trials within 3 months before this study; those who had used other topical treatments for psoriasis within 7 days before enrollment, or had received biologic therapies in the 3 months before enrollment; and participants with contraindications to the study treatments or medications.

Patients will terminate the participation if the following conditions arise: (1) Misdiagnosed or erroneously included. (2) Unfeasible to determine the efficacy due to taking other medications. (3) Poor compliance. (4) Development of other serious diseases or conception during the trial period. (5) Occurrence of safety concerns and serious adverse events (AEs). (6) The efficacy demonstrated poor results, preventing the trial from proceeding.

### 2.3 Drug preparation

**FIGURE 1:**
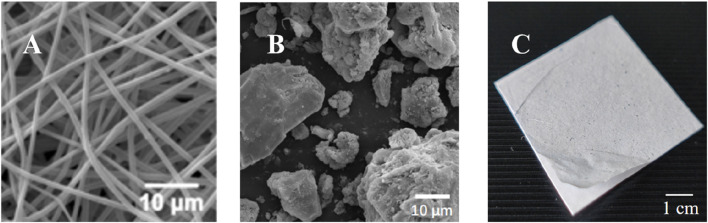
Preparation of IN-PCL/PEO nanofibrous patch. **(A)** PCL/PEO nanofiber membrane image under scanning electron microscopy (SEM). Scale bar: 10 μm. **(B)** Structure of IN drug particles under SEM. Scale bar: 10 μm. **(C)** The image of the finished product of IN electrospun nanofiber membrane. Scale bar: 1 cm.

The electrostatic spinning nanofiber membranes of IN used in this experiment were prepared by the Institute of Basic Theory of Chinese Medicine, China Academy of Traditional Chinese Medicine, and the experimental reagents and experimental apparatus used in the preparation process are listed in Table 1. The photos of the finished product under an electron microscope and the naked eye are listed in Figure 1. Initially, the polymers PCL and PEO were dissolved in hexafluoroisopropanol (1,1,1,3,3,3-Hexafluoro-2-propanol, HFIP) to form an amphiphilic composite nanofibrous membrane (PCL/PEO) for use as a topical drug delivery carrier. Subsequently, the IN powder was incorporated into the electrospinning solution for drug loading. The prepared electrospun solution was spun to obtain the IN electrospun nanofiber membrane. The spinning parameters were as follows: 5% PCL/PEO (8:2), applied voltage of 7 kV, flow rate of 0.15 mm/min, jet distance of 12 cm, needle inner diameter of 0.5 mm, temperature of 30°C, humidity of 26%, and a drug loading concentration of 15% for IN (every 100 g of the PCL/PEO contains 15 g of IN). The current batch of Indigo Naturalis was quantitatively analyzed by high-performance liquid chromatography (HPLC), revealing a composition of 2.22% ± 0.46% indigo, 0.33% ± 0.02% indirubin, and 0.03% ± 0.01% tryptanthrin. In preclinical studies, using histopathology, immunohistochemistry, and evaluations for psoriasis, we investigated the anti-psoriasis effect of IN-PCL/PEO nanofibrous patches at the tissue and animal levels. Following that, we conducted preliminary investigations involving three psoriasis patients to assess further the feasibility of applying the IN-PCL/PEO nanofibrous patches to human skin. All three participants exhibited a significant reduction in PASI scores, and no local irritation or discomfort was reported throughout the treatment period. Additionally, no dark stains were observed on their skin or clothing.

**TABLE 1:** Composition and proportion of IN-PCL/PEO nanopatch.

Reagent	Brand	Proportion
Indigo naturalis (IN)	Juyaotang of Anguo City	13%
polycaprolactone (PCL)	Sigma-Aldrich	70%
polyethylene oxide (PEO)	Sigma-Aldrich	17%

### 2.4 Intervention

In the test group, the IN-PCL/PEO nanofibrous patch was applied to the lesions for 30 min once daily, delivering an approximate dose of 0.33 mg/cm^2^ of IN per application. This dosing regimen was optimized based on systematic preclinical investigations from our prior work (Wang et al., 2023; Guo et al., 2022; Li et al., 2021). Specifically, electrospinning solutions with varying IN concentrations (5%, 10%, 15%, 20%, 25%, 30%, and 50%) were tested. Morphological analyses via Scanning electron microscopy revealed that IN concentrations exceeding 15% caused fiber aggregation and reduced homogeneity due to limited solubility. Pharmacodynamic studies in imiquimod (IMQ)-induced psoriasis mice demonstrated that 15% IN-PCL/PEO achieved maximal suppression of epidermal hyperplasia (50.5% reduction vs. IMQ model, *p* < 0.005), inflammatory infiltration, and angiogenesis, comparable to 20% IN-PCL/PEO but with superior cost-effectiveness.

In the control group, topical calcipotriol ointment was applied once daily at 2 mg/cm^2^, this dose corresponds to 0.1 mg/cm^2^ of active calcipotriol, consistent with clinical practice. The dose discrepancy was due to differences in drug delivery mechanisms: The amphiphilic PCL/PEO matrix enables sustained release, whereas calcipotriol requires higher initial concentrations for immediate bioavailability.

### 2.5 Measurements and outcomes

The primary efficacy outcome in this study was the change in the PASI score (unit: points) after 4 weeks of treatment (Perez-Chada et al., 2020). Secondary outcomes included the change in Patient Global Assessment (PGA) (Gollins and Coates, 2023; Yu et al., 2023), subjective symptom assessments, skin lesion irritation response score, safety, and treatment satisfaction. The PGA was assessed using the five-point Investigator’s Global Assessment (IGA) Scale (0 = clear, 1 = almost clear, 2 = mild, 3 = moderate, 4 = severe), evaluating erythema, scaling, and induration of psoriatic lesions, adapted from the Psoriasis Area and Severity Index (PASI). These scales have been validated for strong criterion validity, and previous studies have shown a significant correlation with PASI scores (Yu et al., 2023; Strober et al., 2019).

To ensure the objectivity of primary and secondary endpoints, three independent dermatologists performed all clinical evaluations under blinded conditions. Treatment group assignments and lesion-side allocations were strictly concealed from evaluators, who underwent standardized training aligned with EuroGuiDerm guidelines (Nast et al., 2021). During patient self-assessment of satisfaction, specific formulation details were withheld from participants to minimize subjective bias.

### 2.6 Safety assessment

Before initiating the trial, routine laboratory tests were performed, including complete blood count, urinalysis, liver function tests, and renal function assessments (Armstrong et al., 2023). The primary focus of these tests was to monitor specific indicators for abnormalities. The complete blood count was conducted to evaluate the number and status of red blood cells, white blood cells, platelets, and other related parameters, providing an overall assessment of the patient’s hematologic health. Urinalysis was performed to detect abnormal components in the urine and to assess renal function and the overall health of the urinary system. Liver function tests were conducted to evaluate liver metabolism and detoxification functions, including measurements of aspartate aminotransferase (AST), alanine aminotransferase (ALT), total bilirubin, and other relevant biomarkers. Renal function was evaluated through serum creatinine and blood urea nitrogen indicators, which reflect the kidneys’ filtration and excretion capacity.

In addition, the occurrence of adverse reactions during drug administration, such as edema, pain, burning sensation, and exacerbation of itching, was closely monitored. In the event of any adverse reactions, appropriate symptomatic treatment was promptly administered, and follow-up was conducted for an additional 2 weeks after resolution of the responses. An evaluation was performed to determine whether continuation of the investigational drug was appropriate. The onset time, potential causes, and corresponding treatment measures for any adverse reactions were meticulously recorded. To standardize and guide the assessment of skin irritation, a rating scale for skin lesion irritation reactions (Table 2) was developed based on the Expert Consensus on the Evaluation of Topical Adverse Reactions to Topical Dermal Drugs.

**TABLE 2:** Skin lesion irritation response rating scale.

Scale	Subjective evaluation Pain, burning sensation	Objective evaluation Papules, edema, blisters, macules, exudates, pustules, vesicles, exudates and ulcers, hypertrophy, desquamation
0	None	None
1	Mild, does not affect daily life and sleep	Mild, no edema (lesions not palpable) and papules
2	Moderate, interferes with daily life but not sleep	Moderate, with edema (lesions are palpable) and papules
3	Severe, interferes with sleep	Severe, with blisters, macules, oozing or pustules, vesicles, oozing or ulcers or windburn, hypertrophy

Safety monitoring was extended beyond the 4-week treatment period to include a 2-week post-treatment follow-up phase. Participants were instructed to report any delayed adverse events (AEs) via scheduled telephone interviews at weeks five and 6. AEs were categorized based on the Common Terminology Criteria for Adverse Events (CTCAE), and severity grades (mild, moderate, severe) documented. For any AE ≥ Grade 2 (moderate), the predefined protocol mandated immediate discontinuation of the intervention, initiation of symptomatic management (e.g., topical corticosteroids for dermatitis), and referral to a dermatologist if unresolved within 48 h. All AEs were recorded regardless of causality attribution.

### 2.7 Sample size

The sample size was calculated based on a two-tailed paired *t*-test to detect a clinically meaningful difference in PASI scores between the IN-PCL/PEO nanofibrous patch and calcipotriol ointment. The minimal clinically important difference (MCID) of 1.2 points in PASI score was predetermined through a Delphi consensus process involving eight psoriasis specialists based on clinical practice, while the standard deviation (SD) of 2.3 points was derived from pilot data (Wang et al., 2023). With a two-side significance level of α = 0.05 and power of 80%, we obtained a minimum sample size of 30 cases per group. Considering the dropout rate of 20%, 36 participants were initially enrolled.

### 2.8 Randomization

This study adopted a randomized envelope method to allocate 36 patients with psoriasis vulgaris into Group 1 and Group 2 in a 1:1 ratio. A total of 36 envelopes were prepared by the researcher, with the following treatment assignments: envelopes numbered 01 to 18 contained assignments of “left A” (IN-PCL/PEO nanofibrous patch) and “right B” (calcipotriol ointment). In contrast, envelopes numbered 19 to 36 contained “left B” and “right A” assignments. This design ensured alternating treatments for the left and right skin lesions. IN-PCL/PEO nanofibrous patches were prepared and supplied by the Institute of Basic Research, China Academy of Traditional Chinese Medicine, while the calcipotriol ointment (30 mL:1.5 mg) was provided by LEO Laboratories Limited, Ireland.

### 2.9 Statistical analysis

Statistical analyses were performed using SPSS 26.0. Continuous variables were analyzed via paired t-tests (normally distributed) or Wilcoxon signed-rank tests (nonparametric). For endpoints involving multiple comparisons (e.g., weekly PASI scores, PGA, erythema, scaling), Holm-Bonferroni correction was applied to control the family-wise error rate at α = 0.05. Effect sizes (Cohen’s *d*) with 95% confidence intervals (*CI*) were calculated to assess clinical relevance.

## 3 Results

### 3.1 Participant flow

**FIGURE 2:**
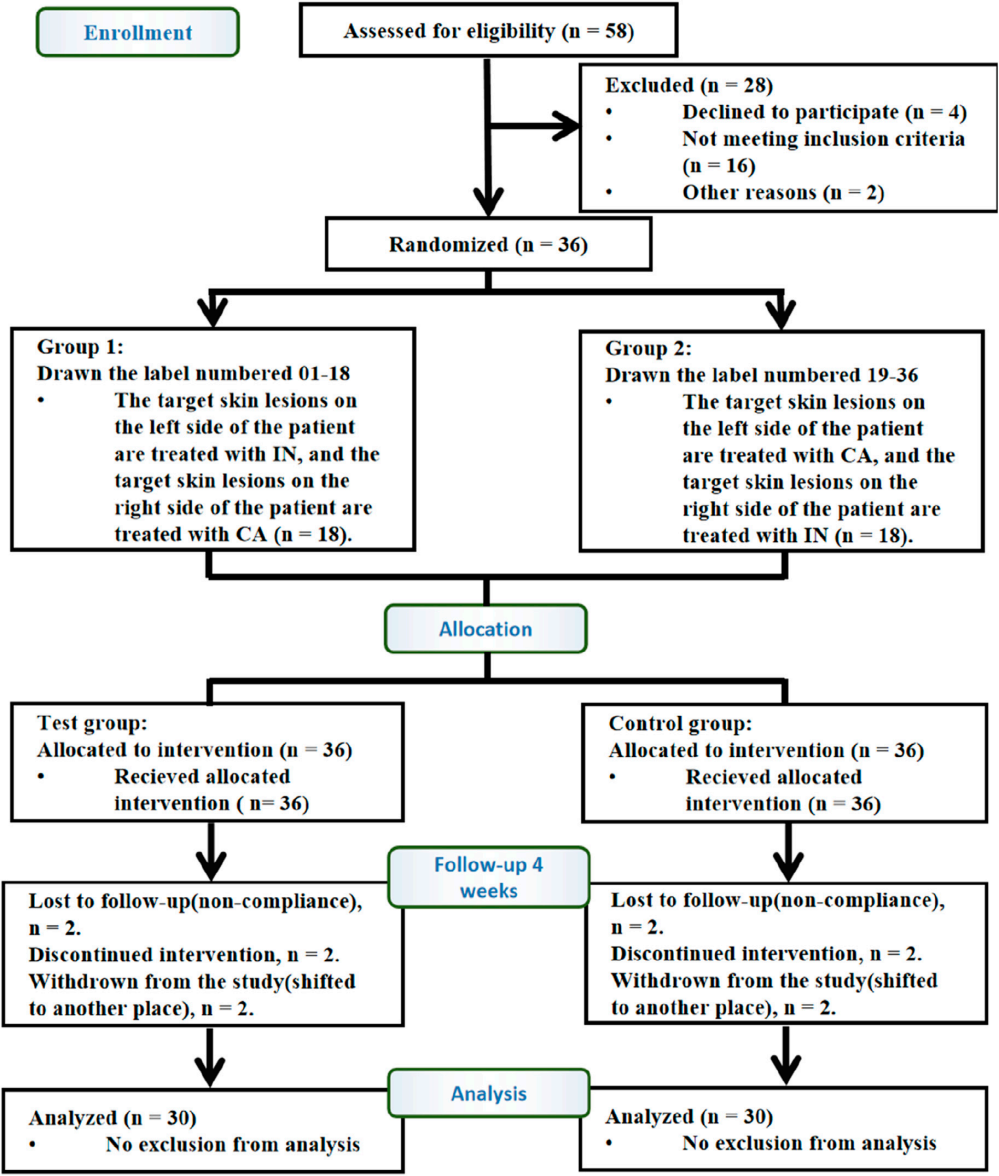
Flow diagram of the trial design. A total of 58 patients were diagnosed with vulgaris psoriasis, of which 16 did not meet the inclusion criteria, four declined to participate in the trial, and two refused to participate for other reasons. Therefore, 36 cases were included. Participants were randomly assigned in a 1:1 ratio to the control group (36) and test group (36). Finally, 30 participants completed 4 weeks of intervention. Paired design: Each participant received both IN-PCL/PEO patches (test) and calcipotriol ointment (control) on contralateral lesions. Abbreviations: IN, IN-PCL/PEO nanofibrous patch; CA, calcipotriol ointment.

A total of 36 patients with psoriasis were enrolled in the clinical trial. Of these, 36 patients who met the inclusion criteria were randomly assigned to the test and control groups in a 1:1 ratio. Thirty participants completed the study, while six patients withdrew. Among the six withdrawals, two patients were unable to return to the clinic due to scheduling conflicts, two discontinued due to unsatisfactory treatment efficacy, and two were unable to complete the study due to the use of other medications that interfered with the efficacy assessment. Statistical analysis was conducted based solely on the data from the 30 participants who completed the study (Figure 2).

### 3.2 Clinico-demographic profile

This study enrolled 30 patients, comprising 21 males and nine females, with an age distribution from 18 to 65 years, encompassing young, middle-aged, and elderly demographic groups. The cohort consisted of seven young patients, 15 middle-aged patients, and eight elderly patients. The disease duration varied significantly among participants, ranging from 1 to 20 years, with 12 patients presenting in the progressive stage and 18 in the quiescent stage of psoriasis. Regarding disease severity, 11 patients were classified as having mild to moderate psoriasis, while 19 exhibited moderate psoriasis. As this investigation was conducted as a semi-controlled trial, no statistically significant differences were observed in these baseline characteristics between the treatment groups, ensuring comparability across study arms.

### 3.3 Change in PASI score

Baseline-adjusted mean PASI scores (unit: points) were 6.30 ± 2.32 (test group) and 6.23 ± 2.27 (control group). Following 4-week treatment, both groups demonstrated significant improvements: the scores decreased from 6.30 ± 2.32 to 2.13 ± 1.57 in the test group (*p* < 0.001), and from 6.23 ± 2.27 to 2.23 ± 1.50 in the control group (*p* < 0.001), with ANCOVA-adjusted differences remaining statistically significant. However, intergroup comparison revealed that the mean difference in PASI reduction was merely −0.10 ± 0.89 (Cohen’s *d* = 0.11, 95%*CI*: 0.43 to 0.23), which was not statistically significant (*p* = 0.541). Consequently, both therapeutic regimens can be considered equally effective in improving PASI scores. After 4 weeks of treatment, scores significantly decreased to 2.13 ± 1.57 and 2.23 ± 1.50 (Table 3), respectively (*p* < 0.001).

**TABLE 3:** Change in PASI score from baseline to final follow-up.

Follow-ups	Test group	Control group	$\bar{d}$ ± s	Effect size (Cohen’s d, 95% CI)	P-value
Baseline (D0)	6.30 ± 2.23	6.23 ± 2.27	0.07 ± 0.25	0.28 (−0.14–0.70)	0.161
Follow-up1 (D7)	5.43 ± 1.63	4.50 ± 1.68	0.93 ± 0.79	1.18 (0.71–1.65)	<0.001
Follow-up2 (D14)	3.97 ± 1.38	3.57 ± 1.52	0.40 ± 0.90	0.44 (0.02–0.86)	0.021
Follow-up3 (D21)	2.87 ± 1.46	2.43 ± 1.55	0.43 ± 0.86	0.50 (0.08–0.92)	0.010
Follow-up4 (D28)	2.13 ± 1.57	2.23 ± 1.50	−0.10 ± 0.89	0.11 (−0.43 to 0.23)	0.541
‾d ± s (D28-D0)	4.20 ± 1.95	4.00 ± 1.89	-	-	-
P-value	<0.001	<0.001	-	-	-

Abbreviations: PASI, psoriasis area and severity index; d ± s, baseline-adjusted mean difference ±SD; *P*-values derived from paired t-tests with Holm-Bonferroni correction for multiple comparisons. *CI*, confidence interval; Effect size (Cohen’s *d*) was calculated as the mean difference divided by the standard deviation of differences. The 95% *CI*, was derived from a paired t-distribution with n = 30.

**FIGURE 3:**
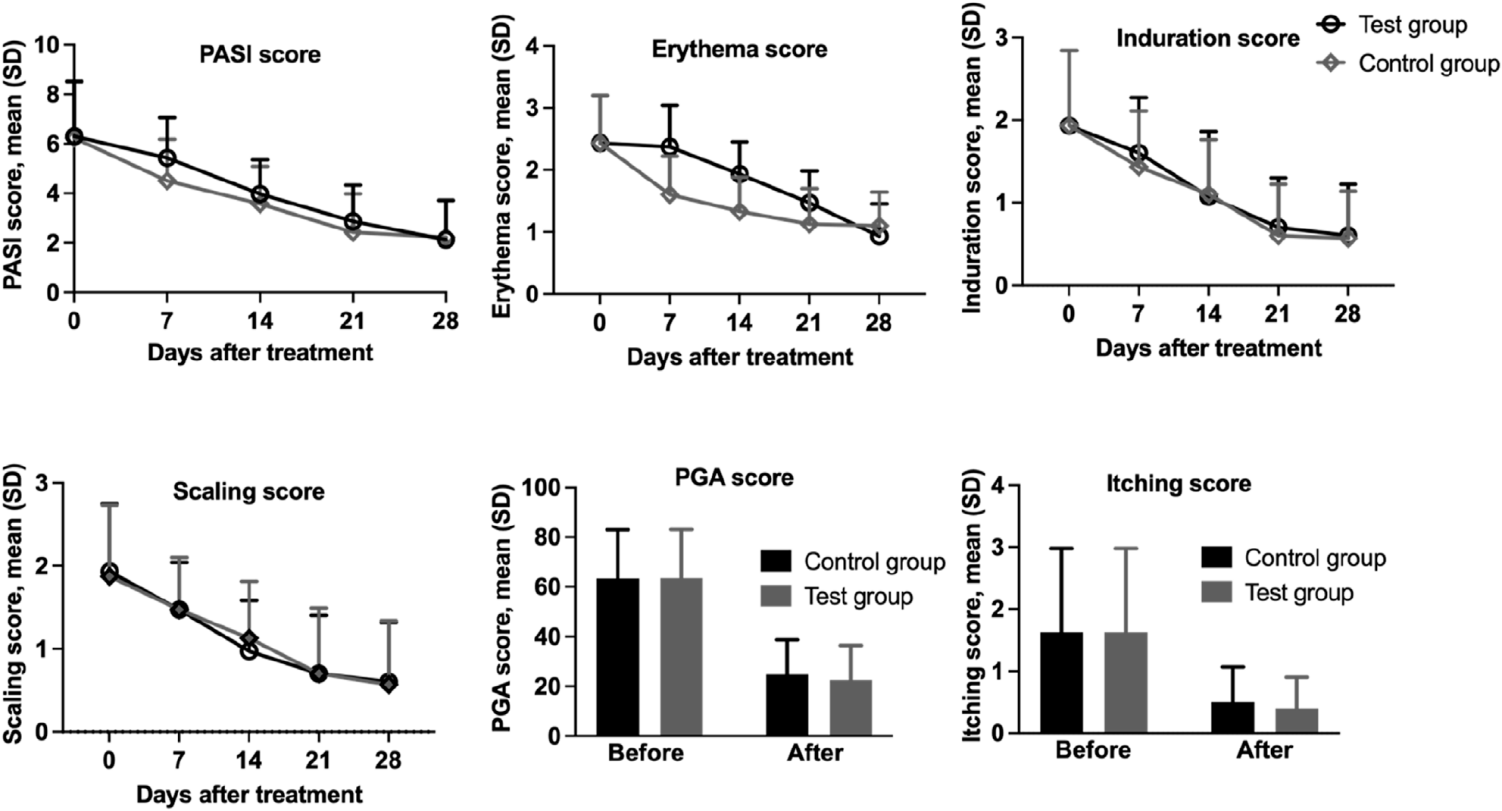
Assessment of PASI score, erythema, infiltration, scaling, PGA, and pruritus in 30 patients with psoriasis treated with IN-PCL/PEO nanofibrous patch and calcipotriol ointment.

Despite no significant difference was observed between the two groups regarding overall PASI score reductions (Figure 3). However, 27 participants (90.0%) in the test group achieved PASI 50, with 43.3% attaining PASI 75. In comparison, 26 participants (86.7%) in the control group achieved PASI 50, while only 33.3% reached PASI 75. Proportions of lesions achieving PASI 50 and PASI 75 were compared using McNemar’s test for matched pairs. No significant differences were observed between groups (PASI 50: 90.0% vs. 86.7%, *p* = 0.774; PASI 75: 43.3% vs. 33.3%, *p* = 0.453).

### 3.4 Improvement in patient’s global assessment (PGA)

The PGA score in both the test group and the control group showed significant reductions, decreasing significantly from 2.54 ± 0.78 and 2.53 ± 0.79 at baseline to 0.90 ± 0.56 and 0.99 ± 0.56 points after the completion of the trial, respectively (*p* < 0.001). No statistically significant difference was observed between the test and control groups (*p* = 0.050), indicating that both interventions were equally effective in improving PGA scores.

### 3.5 Change in subjective parameters

**FIGURE 4:**
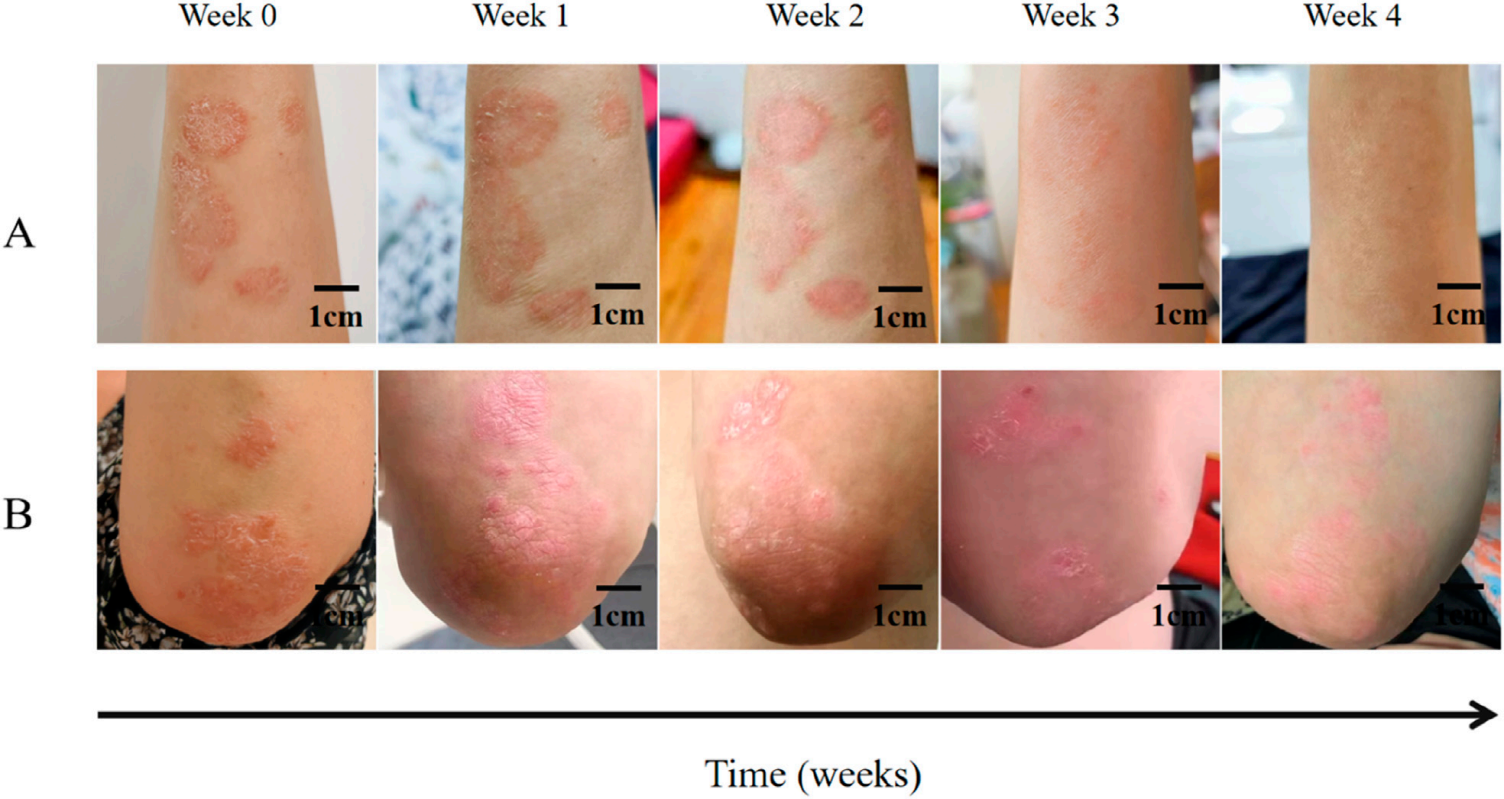
Clinical observation of IN-PCL/PEO nanofibrous patch and calcipotriol ointment in treating a 46-year-old female patient with plaque psoriasis. **(A)** The patient’s psoriatic lesions on her right forearm were treated with indigo nanomembrane for four consecutive weeks. Scale bar: 1 cm. **(B)** The patient’s psoriasis lesions on her left elbow joint were treated with calcipotriol ointment for 4 weeks. Scale bar: 1 cm.

We also separately analyzed the scores of individual skin lesion parameters (erythema, scaling, infiltration, and itching) (Table 4). Both the test and control groups demonstrated statistically significant reductions (*p* < 0.001) in erythema scores from baseline values of 2.43 ± 0.77 to 0.93 ± 0.52 (61.73% reduction) and 1.10 ± 0.54 (54.73% reduction), respectively. Regarding scaling scores, the test group showed a decrease from 1.93 ± 0.82 to 0.60 ± 0.72 (68.91% reduction), while the control group improved from 1.87 ± 0.86 to 0.57 ± 0.77 (69.52% reduction), with both reductions being statistically significant (*p* < 0.001). Similarly, induration scores decreased from 1.93 ± 0.91 to 0.60 ± 0.62 (68.91% reduction) in the test group and to 0.57 ± 0.57 (70.46% reduction) in control group (*p* < 0.001). Itching scores also exhibited marked improvement, declining from baseline values of 1.63 ± 1.35 to 0.40 ± 0.50 (75.46% reduction) in the treatment group and 0.50 ± 0.57 (69.32% reduction) in the control group (*p* < 0.001; Figure 4). These results demonstrate clinical improvement across all measured dermatological parameters in both study arms.

**TABLE 4:** Changes in the severity of psoriasis symptoms from baseline to treatment completion (within group and between group analysis).

Group	Mean rank at baseline	Mean rank at 1st follow-up	Mean rank at 2nd follow-up	Mean rank at 3rd follow-up	Mean rank at 4th follow-up	P-value
Erythema						
Test	2.43	2.37	1.93	1.47	0.93	<0.001
Control	2.43	1.60	1.33	1.13	1.10	<0.001
P-Value	1.000	<0.001	<0.001	0.002	0.057	-
Induration						
Test	1.93	1.60	1.07	0.70	0.60	<0.001
Control	1.93	1.43	1.10	0.60	0.57	<0.001
P-Value	1.000	0.096	0.787	0.184	0.573	-
Scaling						
Test	1.93	1.47	0.97	0.70	0.60	<0.001
Control	1.87	1.47	1.13	0.70	0.57	<0.001
P-Value	0.161	1.000	0.169	1.000	0.662	-
Itching						
Test	1.63	-	-	-	0.40	<0.001
Control	1.63	-	-	-	0.50	<0.001
P-Value	1.000	-	-	-	0.083	-

### 3.6 Skin lesion irritation response scores and adverse events

The skin lesion irritation response scores were assessed for both the test group and control group, respectively, at the end of the treatment cycle, and the results showed that the irritation response score of the test group was 0.03 ± 0.18, while in the control group, it was 0.50 ± 0.57. The skin lesion irritation response in the test group was significantly lower than in the control group (*p* < 0.001). No severe AEs (Grade ≥3) or delayed AEs were reported during the 4-week treatment or the 2-week post-treatment follow-up period. Two participants in the control group experienced transient burning sensations (Grade 1, localized to the application site), which resolved spontaneously within 24 h without intervention. All safety parameters (blood counts, liver/kidney function) remained within clinically normal ranges throughout the study. Adherence to the AE management protocol was confirmed, with no cases requiring discontinuation or specialist referral. Routine laboratory tests (blood, urine, hepatic/renal function) performed pre- and post-treatment revealed no abnormalities in any of the 30 patients. Additionally, no significant adverse reactions occurred during the entire treatment cycle.

### 3.7 Satisfaction evaluation

At the end of the treatment cycle, a satisfaction score was performed on both the test and control groups, evaluating three aspects: efficacy, skin feel, and staining, with a maximum possible score of 10 points. The results showed that the test group received scores of 8.50 ± 1.48 for efficacy, 9.70 ± 0.60 for skin feel, and 10.00 ± 0.00 for staining. In comparison, the control group scored 8.27 ± 1.61 for efficacy, 8.40 ± 1.40 for skin feel, and 10.00 ± 0.00 for staining. The test group demonstrated a significantly better skin feel score than the control group (*p* < 0.001), indicating superior patient satisfaction with the formulation’s texture.

## 4 Discussion

### 4.1 Interpretation

This study compared the efficacy and safety of calcipotriol ointment, a conventional therapy for chronic plaque psoriasis, with our newly developed IN-PCL/PEO nanofibrous patch. As a novel topical treatment, the IN-PCL/PEO nanofibrous patch demonstrated promising results across various outcome indicators. It not only exhibited comparable therapeutic efficacy to calcipotriol ointment but also showed superior safety. The differences in primary and secondary outcome indicators before and after treatment fell within clinically significant ranges. These findings suggest that IN-PCL/PEO nanofibrous patches could represent a viable topical alternative for psoriasis management, particularly in patients prioritizing tolerability and ease of use.

Although no statistically significant between-group difference was observed in PASI reduction, the narrow confidence interval for the PASI difference (−0.43 to 0.23) indicates that the study was sufficiently powered to exclude the predefined clinically relevant difference (MCID ≥1.2). This supports the conclusion that the IN-PCL/PEO patch and calcipotriol ointment have comparable efficacy in reducing PASI scores over a 4-week period. Future trials with larger cohorts and extended follow-up durations are warranted to confirm these findings and explore potential subtle differences in long-term outcomes.

### 4.2 Mechanistic insights of IN-PCL/PEO nanofibrous patch for psoriasis

The therapeutic efficacy of the IN-PCL/PEO nanofibrous patch in treating chronic plaque psoriasis is attributed to its innovative transdermal delivery system. This system overcomes the limitations of traditional Indigo naturalis (IN) formulations while enhancing the pharmacological effects of its primary active components. We conducted preclinical studies to optimize the dosage regimen and application strategy for the IN-PCL/PEO nanofibrous patch, aiming to strike a balance between therapeutic efficacy and practicality. The optimized dosage was determined to be 15% IN at 0.33 mg/cm^2^ in the patch, with a daily application duration of 30 min (Wang et al., 2023). Initially, we fabricated PCL/PEO nanofibrous membranes containing IN concentrations ranging from 5% to 50% using electrospinning. Scanning electron microscopy (SEM) analyses revealed that concentrations of IN above 15% led to the precipitation of drug particles on the fibers and uneven drug distribution. Further evaluations were performed on imiquimod (IMQ)-induced psoriatic mice, which demonstrated that the 15% IN-PCL/PEO patch effectively suppressed epidermal hyperplasia, resulting in a 50.5% reduction in thickness compared to the model group (*p* < 0.005). Additionally, it decreased inflammatory infiltration and angiogenesis. The therapeutic effect of the 15% IN patch was comparable to that of the 20% IN patch, while being more cost-effective, making it a suitable choice for clinical applications.

The 30-min application duration for the IN-PCL/PEO patch was determined based on the release profiles, the skin permeation and retention of the three active components. *In vitro* drug release studies showed initial burst releases of indirubin (46.9%), tryptanthrin (66.4%), and indigo (8.5%) within 30 min, followed by sustained release phases. This biphasic “burst-sustained” release profile provides therapeutic effects from indirubin and tryptanthrin during short-term application while reducing skin staining from indigo. *Ex vivo* retention studies using Franz diffusion cells confirmed that the 30-min application optimized the transdermal delivery of indirubin (epidermis: 64.0 ± 15.1 ng/cm^2^; dermis: 68.3 ± 20.1 ng/cm^2^) and tryptanthrin (epidermis: 10.4 ± 2.5 ng/cm^2^; dermis: 6.0 ± 0.8 ng/cm^2^), while significantly reducing the retention of indigo (epidermis: 1.4 ± 1.0 ng/cm^2^; dermis: undetectable). In contrast, a lower amount of drugs was detected after applying the traditional IN ointment. Specifically, the level of indirubin was found at 17.4 ± 7.3 ng/cm^2^ in the epidermis and was undetectable in the dermis; tryptanthrin was present at 4.7 ± 1.9 ng/cm^2^ in the epidermis and 5.3 ± 0.67 ng/cm^2^ in the dermis; and indigo was also detected at 17.4 ± 7.3 ng/cm^2^ in the epidermis, but was undetectable in the dermis. This selective drug delivery eliminated the skin staining caused by indigo from IN and enhanced the effective penetration of the main pharmacologically active molecules, indirubin and tryptanthrin. As a result, applying the IN patch for 30 min led to improved drug absorption through the skin permeation and retention.

The underlying mechanism of the patch’s efficacy was further explored in a preclinical study. BALB/c mice (male, 6–8 weeks old) with IMQ-induced psoriatic lesions were randomized into control, model, and six treatment groups. These treatment groups include PCL/PEO, 5% IN-PCL/PEO, 10% IN-PCL/PEO, 15% IN-PCL/PEO nanofibrous patches, an IN ointment, and clobetasol propionate ointment as a positive control, with six mice per group (n = 6). Topical patch applications were performed for 30 min daily over 7 days. The severity of the lesions was assessed through blinded scoring of erythema (0–4), scaling (0–4), and measurements of epidermal thickness using optical coherence tomography (OCT). The results indicated that the 15% IN-PCL/PEO patch significantly inhibited epidermal hyperplasia, resulting in a 50.5% ± 6.6% reduction in thickness. Histopathological analyses revealed a 62% decrease in Ki67-positive proliferating keratinocytes, alongside a reduction of CD68-positive macrophage infiltration and lower CD31-related pathological vascular density. The efficacy of the 15% IN-PCL/PEO patch was comparable to that of clobetasol propionate and was more effective than that of the IN ointment. Notably, there was no skin staining or systemic toxicity observed in the groups treated with the IN-PCL/PEO nanofibrous patches. Meanwhile, the pathological examinations of the liver, spleen and kidney indicated good biocompatibility of the IN patches. These results are consistent with the reported pharmacological properties of the main active components in IN. Tryptanthrin inhibits proliferation of keratinocytes by inducing cell cycle arrest at the G0/G1 phase and also suppresses angiogenesis (Wang et al., 2023), while indirubin modulates TAK1 pathways to reduce inflammation (Zhao et al., 2021).

Results from animal studies led to implementation of a 30-min short-contact regimen in clinical settings, aimed at enhancing patient adherence while ensuring effectiveness. Calcipotriol ointment requires a higher initial drug loading of 2 mg/cm^2^ ointment to deliver 0.1 mg/cm^2^ of active calcipotriol, which is necessary for sustaining its therapeutic efficacy. In contrast, the IN-PCL/PEO patch features an amphiphilic matrix that allows for a lower loading dose of IN (0.33 mg/cm^2^), enabling an even smaller amount of active components to be incorporated into nanosized fibers. This design facilitates the rapid release of active components, indirubin and tryptanthrin, while promoting efficient skin permeation. As a result, it minimizes the risk of systemic exposure and reduces the time required for application.

Due to ethical constraints, direct biomarker assessments in patients were not feasible. However, the absence of skin staining and systemic toxicity, when combined with preclinical studies, suggests that the IN-PCL/PEO patch can effectively release and enhance the skin permeation of indirubin and tryptanthrin while minimizing the release of indigo. This innovative drug delivery mechanism appears to specifically target key pathological features of psoriasis, including keratinocyte hyperproliferation, inflammation, and angiogenesis (Wang et al., 2023).

### 4.3 Overall evidence

Previous studies have suggested that the therapeutic effects of IN on psoriasis may be primarily attributed to its active components, tryptanthrin, and indirubin (Xie et al., 2018; Lu et al., 2022; Xue et al., 2018). Based on existing literature, these effective biological components exert anti-psoriasis effects through multiple mechanisms, including the inhibition of angiogenesis, inhibition of keratinocyte proliferation, anti-inflammation, and immune regulation.

Current evidence indicates that tryptanthrin may influence angiogenesis-related gene expression by suppressing angiotensinogen promoter activity. In vitro studies demonstrate that tryptanthrin potentially induces cell cycle arrest at the G2/M phase, preventing further cell division and directly reducing cell proliferation rates (Xiong et al., 2023; Chang et al., 2015). In addition, research suggests tryptanthrin may also exerts its role by inhibiting the Akt and FAK signaling pathways. The Akt signaling pathway is an important regulatory pathway for cell survival and proliferation, while the FAK signaling pathway is the key to regulating cell adhesion, migration, and survival. The inhibition of these two signal pathways by tryptanthrin together inhibits the proliferation and migration of vascular endothelial cells. Furthermore, Cheng et al. (2020) proposed that IN and its active component, tryptanthrin, might exhibit anti-inflammatory effects by downregulating the expression of multiple cytokines required for Th17 polarization.

As another key component, literature indicates that indirubin may affect keratinocyte function through regulation of Wnt/β-catenin signaling. Some studies report that indirubin may inhibit the expression of transglutaminase 1 (TGase1) and downregulates the expression of cyclin-dependent kinase 1 (CDK1), thereby suppressing the proliferation of keratinocytes (Blazevic et al., 2015; Liu et al., 2020; Sekhon and Koo, 2018). In animal models, indirubin has shown potential to ameliorate cutaneous inflammation, possibly through modulation of MAPK activity. Other research data indicate (Chuang et al., 2024; Kim et al., 2013) that indirubin may influence serum immunoglobulin E (IgE) levels and cytokine production in a dose-dependent manner by modulating Th1- and Th2-mediated immune responses. Studies also observed its regulatory effects on nuclear factor κB (NF-κB), IκB-α and MAPK expression. Notably, data suggest indirubin may selectively increase the numbers of CD4^+^, CD25^+^ and regulatory T (Treg) cells, which could be a potential mechanism for its promotion of immune balance.

The enhanced therapeutic outcomes observed with the IN-PCL/PEO nanofibrous patch can be mechanistically attributed to its optimized transdermal delivery system. Unlike traditional ointments, which suffer from poor drug penetration due to the stratum corneum barrier and formulation viscosity, the amphiphilic PCL/PEO nanofibers provide a high surface area-to-volume ratio and interconnected porous structure. This design facilitates sustained release of IN’s active compounds (e.g., tryptanthrin and indirubin) into the epidermal and dermal layers, as evidenced by our preclinical pharmacokinetic data (Wang et al., 2023). Specifically, the patch’s burst release within 30 min ensures rapid bioavailability of anti-inflammatory agents (e.g., tryptanthrin), while the sustained release phase maintains therapeutic concentrations of antiproliferative components (e.g., indirubin) over time. This biphasic delivery profile aligns with the dual pathogenesis of psoriasis—acute inflammation and chronic keratinocyte hyperproliferation—thereby enhancing pharmacodynamic synergy compared to conventional ointments. Moreover, our previous research identified indigo as the primary component responsible for the dyeing properties of IN, but it was found to have negligible therapeutic effect on psoriasis. Therefore, during the preparation of the IN-PCL/PEO nanofibrous patch, while this component was incorporated into the formulation, the formulation was designed to ensure that indigo could not effectively be released to the skin surface. This approach made the IN nanomembrane cleaner and more aesthetically pleasing while altering the drug penetration and retention mechanisms compared to traditional IN ointments. This innovation addresses the issues of excessive staining and limited skin penetration associated with IN ointments.

### 4.4 Safety and adverse events

When evaluating the safety of IN-PCL/PEO nanofibrous patch and calcipotriol ointment, we found that the test group showed better safety and tolerability than the control group. Specifically, in the treatment group using IN-PCL/PEO nanofibrous patch, no skin irritation or other related adverse reactions were observed. In contrast, four patients in the control group reported pain and burning at the application site, but these symptoms eased after drug discontinuation. In addition, for specific indicators that may affect liver and kidney function and routine hematuria, no abnormal changes were observed in this study before and after the trial. This finding further supports the safety advantages of the test group. Based on these observations, we can conclude that IN-PCL/PEO nanofibrous patches show high safety and low risk of adverse reactions in the treatment of psoriasis.

### 4.5 Satisfaction evaluation

After the treatment, the patient evaluated three aspects: efficacy, skin feel, and dyeing. The patient was highly satisfied with the IN-PCL/PEO nanofibrous patch, and it performed well in all indicators of efficacy, skin feel, and dyeing. Furthermore, the test group was better than the control group in terms of skin feeling. The above results may be related to the good skin adaptability and convenience of the use of IN-PCL/PEO nanofibrous patches.

### 4.6 Limitations of the study

This study has several limitations that should be acknowledged. First, the open-label design, despite employing a semi-compartmental paired approach to control inter-individual variability, may have introduced bias in subjective endpoints such as PGA and patient satisfaction. Participants’ awareness of receiving a novel nanofibrous patch versus a conventional ointment could have influenced their perception of treatment outcomes. To mitigate this, we implemented blinded evaluations by three independent dermatologists trained in standardized PASI and PGA scoring (aligned with EuroGuiDerm guidelines (Nast et al., 2021)), and masked treatment labels during patient satisfaction surveys. However, inherent differences in the physical properties of the interventions (e.g., patch adhesion vs. ointment texture) might still affect subjective responses. Future trials should adopt double-blind designs with placebo-matched formulations to further minimize bias.

Second, the generalizability of findings is constrained by several methodological limitations. The modest sample size (n = 36 enrolled, n = 30 completed) and short-term follow-up (4 weeks) restrict robust conclusions regarding long-term efficacy and safety, particularly for detecting rare adverse events. These limitations are compounded by the single-center design and homogeneous population, which may not fully represent the broader psoriasis demographic. While preliminary results demonstrated clinically meaningful PASI reductions in both groups (test group: Δ4.20 ± 1.95; control group: Δ4.00 ± 1.89), confirmatory studies with larger cohorts and extended observation periods are imperative to validate therapeutic durability. Furthermore, the 4-week trial duration may have attenuated the sensitivity of PGA and subjective symptom scales to detect incremental improvements, especially in patients with milder baseline severity. Notably, however, significant reductions in key parameters (e.g., erythema: 61.7% vs. 54.7%) suggest retained responsiveness of these scales to short-term changes. To enhance reliability, future investigations should integrate objective measures (e.g., spectrophotometric erythema index) alongside subjective assessments, thereby addressing both clinical and methodological dimensions of outcome evaluation.

In addition, the team conducted major research on the effect of IN-PCL/PEO nanofibrous patches on pathological changes such as inflammation, keratinocyte proliferation, and vascular proliferation in psoriasis model mice, more in-depth investigations on the molecular mechanism behind required to be strengthened.

### 4.7 Clinical implications

This study highlights the transformative potential of nanotechnology in bridging traditional herbal medicine with modern therapeutic demands. The IN-PCL/PEO patch not only preserves the bioactive properties of IN but also overcomes the practical limitations of its semisolid formulations. By eliminating skin staining and reducing irritation, this innovation addresses two major barriers to patient adherence in chronic psoriasis management. In contrast to calcipotriol ointment—which requires meticulous application to avoid perilesional skin damage—the patch’s compartmentalized design enables targeted drug delivery with minimal handling errors. These advantages may translate to higher treatment satisfaction and lower healthcare costs due to reduced need for rescue therapies or dose escalations. For clinicians, the IN-PCL/PEO patch offers a steroid-free alternative with a favorable safety profile, particularly suitable for long-term use in pediatric or elderly populations vulnerable to topical corticosteroid side effects.

## 5 Conclusion

This study demonstrated the potential of IN-PCL/PEO nanofibrous patches in alleviating symptoms of psoriasis vulgaris over a 4-week period. Our findings suggest that the IN-PCL/PEO patches exhibit comparable efficacy to calcipotriol ointment in reducing PASI scores, with additional advantages in patient comfort and tolerability. While these results are promising, the modest sample size and short-term follow-up necessitate cautious interpretation. Future studies with larger cohorts and extended observation periods are required to confirm these preliminary findings and evaluate long-term safety and durability of treatment effects.

Our results indicated that during the 4-week intervention period, the IN-PCL/PEO nanofibrous patches were associated with reductions in PASI scores, suggesting potential improvements in symptom relief and lesion area. However, these findings should be interpreted with caution given the study’s limited sample size and short-term observation period. The observed therapeutic effects of the IN-PCL/PEO nanofibrous patches may be attributed to the bioactive properties of IN, including anti-inflammatory, antiproliferative, and immunomodulatory mechanisms. However, the similarity in PASI reduction between groups underscores the need for further mechanistic studies to delineate the unique contributions of the nanofiber delivery system.

While the therapeutic effects of IN-PCL/PEO nanofibrous patches did not significantly exceed those of calcipotriol ointment in specific psoriasis-related parameters, they demonstrated superior patient experience and skin irritation performance. This suggests that the study underscores the potential of IN and its active ingredients in treating psoriasis vulgaris, and the advantages of integrating traditional Chinese medicine with modern nanotechnology. IN-PCL/PEO nanofibrous patches present an alternative treatment option for patients who cannot tolerate calcipotriol ointment.

This study represents an innovative approach to developing and applying IN-PCL/PEO nanofibrous patches, being the first of its kind in psoriasis treatment. The development of this nanofiber membrane not only enhances the efficiency of topical drug delivery but also significantly minimizes side effects, providing a novel therapeutic option for psoriasis patients. In addition, this study successfully exemplifies interdisciplinary integration by merging herbal medicine with modern medical technology to explore new therapeutic strategies for psoriasis. Even though there have been numerous reports on nano preparations for psoriasis, applying this technology within the realm of traditional Chinese medicine marks a significant advance. This interdisciplinary approach offers a fresh perspective and methodology for treating psoriasis with traditional Chinese medicine, showcasing the substantial potential of nanotechnology in topical preparations.

## Data Availability

The original contributions presented in the study are included in the article/supplementary material, further inquiries can be directed to the corresponding author/s.

## Abbreviations

AEs, Adverse Events; ALT, Alanine Aminotransferase; AST, Aspartate Aminotransferase; CA, Calcipotriol Ointment; CDK1, Cyclin-dependent kinase 1; CP, Clobetasol Propionate; FAK, Focal Adhesion Kinase; SEM, Scanning electron microscopy; HFIP, 1,1,1,3,3,3-Hexafluoro-2-propanol; HPLC, High Performance Liquid Chromatography; IMQ, Imiquimod; IN, Indigo Naturalis; MAPK, Mitogen-Activated Protein Kinase; MCID, Minimal Clinically Important Difference; PASI, Psoriasis Area and Severity Index; PCL, Poly (ε-caprolactone); PEO, Poly (ethylene oxide); PGA, Patient Global Assessment; TGase1, Transglutaminase 1.

## References

[B1] Arampatzis A. S. Kontogiannopoulos K. N. Theodoridis K. Aggelidou E. Rat A. Willems A. (2021). Electrospun wound dressings containing bioactive natural products: physico-chemical characterization and biological assessment. Biomater. Res. 25 (1), 23. 10.1186/s40824-021-00223-9 34271983 PMC8284004

[B2] Armstrong A. W. Bohannan B. Mburu S. Coates L. C. Ogdie A. Alarcon I. (2023). Patient perspectives on psoriatic disease Burden: results from the global psoriasis and beyond survey. Dermatology 239 (4), 621–634. 10.1159/000528945 37075723 PMC10357389

[B3] Blazevic T. Heiss E. H. Atanasov A. G. Breuss J. M. Dirsch V. M. Uhrin P. (2015). Indirubin and indirubin derivatives for counteracting proliferative diseases. Evid. Based Complement. Altern. Med. 2015, 654098. 10.1155/2015/654098 PMC4589628 26457112

[B4] Caldarola G. De Luca E. Mariani M. Chiricozzi A. Peris K. De Simone C. (2023). Drug survival of methotrexate and predictor factors for discontinuation in psoriasis. Int. J. Dermatol 62 (5), 649–656. 10.1111/ijd.16652 36961109

[B5] Campbell F. Laws P. (2024). Managing risk of liver fibrosis in patients with psoriasis being considered for methotrexate. Br. J. Dermatol 191 (2), 163. 10.1093/bjd/ljae114 38504462

[B6] Chang H. Huang S. T. Yeh Y. C. Wang H. S. Wang T. H. Wu Y. H. (2015). Indigo naturalis and its component tryptanthrin exert anti-angiogenic effect by arresting cell cycle and inhibiting Akt and FAK signaling in human vascular endothelial cells. J. Ethnopharmacol. 174, 474–481. 10.1016/j.jep.2015.08.050 26341616

[B7] Chang H. Yeh Y. C. Chueh H. Y. Pang J. H. S. (2019). The anti-angiogenic effect of tryptanthrin is mediated by the inhibition of apelin promoter activity and shortened mRNA half-life in human vascular endothelial cells. Phytomedicine 58, 152879. 10.1016/j.phymed.2019.152879 31005035

[B8] Cheng H. Kuo Y. Z. Chang C. Y. Chang C. H. Fang W. Y. Chang C. N. (2020). The anti-TH17 polarization effect of Indigo naturalis and tryptanthrin by differentially inhibiting cytokine expression. J. Ethnopharmacol. 255, 112760. 10.1016/j.jep.2020.112760 32173427

[B9] Chiricozzi A. Faleri S. Saraceno R. Bianchi L. Buonomo O. Chimenti S. (2015). Tofacitinib for the treatment of moderate-to-severe psoriasis. Expert Rev. Clin. Immunol. 11 (4), 443–455. 10.1586/1744666X.2015.1013534 25666451

[B10] Chuang H. Chuang K. J. Cheng P. C. Hsieh C. L. Fan Y. Y. Lee Y. L. (2024). Indirubin induces tolerogenic dendritic cells via aryl hydrocarbon receptor activation and ameliorates allergic asthma in a murine model by expanding Foxp3-expressing regulatory T cells. Phytomedicine 135, 156013. 10.1016/j.phymed.2024.156013 39270571

[B11] Dias M. F. R. G. Loures A. El Kadi N. Aranha N. S. Machado A. Fontinha P. R. B. (2023). Trichoscopic pearl: dynamic trichoscopy sequence of scalp psoriasis before and after the development of Auspitz's sign. Arch. Dermatol Res. 316 (1), 39. 10.1007/s00403-023-02788-y 38085350

[B12] Gollins C. E. Coates L. C. (2023). Validation of a new measure of patient global assessment in psoriasis. Br. J. Dermatol 189 (4), 364–365. 10.1093/bjd/ljad228 37410548

[B13] Greb J. E. Goldminz A. M. Elder J. T. Lebwohl M. G. Gladman D. D. Wu J. J. (2016). Psoriasis. Nat. Rev. Dis. Prim. 2, 16082. 10.1038/nrdp.2016.82 27883001

[B14] Griffiths C. E. Barker J. N. (2007). Pathogenesis and clinical features of psoriasis. Lancet 370 (9583), 263–271. 10.1016/S0140-6736(07)61128-3 17658397

[B15] Guo S. Wang P. Song P. (2022). Electrospinning of botanicals for skin wound healing. Front. Bioeng. Biotechnol. 10, 1006129. 10.3389/fbioe.2022.1006129 36199360 PMC9527302

[B16] Han Y. Wei H. Ding Q. Ding C. Zhang S. (2024). Advances in electrospun nanofiber membranes for dermatological applications: a review. Molecules 29 (17), 4271. 10.3390/molecules29174271 39275118 PMC11396802

[B17] Hwang J. K. Ricardo J. W. Lipner S. R. (2023). Efficacy and safety of Nail psoriasis targeted therapies: a systematic review. Am. J. Clin. Dermatol 24 (5), 695–720. 10.1007/s40257-023-00786-4 37209391

[B18] Kielbowski K. Bakinowska E. Bratborska A. W. Pawlik A. (2024). The role of adipokines in the pathogenesis of psoriasis - a focus on resistin, omentin-1 and vaspin. Expert Opin. Ther. Targets 28 (7), 587–600. 10.1080/14728222.2024.2375373 38965991

[B19] Kim M. H. Choi Y. Y. Yang G. Cho I. H. Nam D. Yang W. M. (2013). Indirubin, a purple 3,2- bisindole, inhibited allergic contact dermatitis via regulating T helper (Th)-mediated immune system in DNCB-induced model. J. Ethnopharmacol. 145 (1), 214–219. 10.1016/j.jep.2012.10.055 23149289

[B20] Li N. Qin Y. Dai D. Wang P. Shi M. Gao J. (2021). Transdermal delivery of therapeutic compounds with Nanotechnological Approaches in psoriasis. Front. Bioeng. Biotechnol. 9, 804415. 10.3389/fbioe.2021.804415 35141215 PMC8819148

[B21] Liu S. G. Luo G. P. Qu Y. B. Chen Y. F. (2020). Indirubin inhibits Wnt/β-catenin signal pathway via promoter demethylation of WIF-1. BMC Complement. Med. Ther. 20 (1), 250. 10.1186/s12906-020-03045-9 32795328 PMC7427955

[B22] Lu X. Wang H. Wang H. Xie F. Jiang C. Shen D. (2022). Indirubin combined with umbilical cord mesenchymal stem cells to relieve psoriasis-like skin lesions in BALB/c mice. Front. Immunol. 13, 1033498. 10.3389/fimmu.2022.1033498 36466901 PMC9709816

[B23] Messana J. M. Rocher L. L. Ellis C. N. Fradin M. S. VanGurp J. R. Cantu-Gonzalez G. (1990). Effects of cyclosporine on renal function in psoriasis patients. J. Am. Acad. Dermatol 23 (6 Pt 2), 1288–1291. 10.1016/0190-9622(90)70356-m 2277137

[B24] Nast A. Smith C. Spuls P. I. Avila Valle G. Bata-Csörgö Z. Boonen H. (2021). EuroGuiDerm Guideline on the systemic treatment of Psoriasis vulgaris - Part 2: specific clinical and comorbid situations. J. Eur. Acad. Dermatol Venereol. 35 (2), 281–317. 10.1111/jdv.16926 33547728

[B25] Nowowiejska J. Baran A. Flisiak I. (2021). Aberrations in lipid expression and metabolism in psoriasis. Int. J. Mol. Sci. 22 (12), 6561. 10.3390/ijms22126561 34207318 PMC8234564

[B26] Parisi R. Iskandar I. Y. K. Kontopantelis E. Augustin M. Griffiths C. E. M. Ashcroft D. M. (2020). National, regional, and worldwide epidemiology of psoriasis: systematic analysis and modelling study. BMJ 369, m1590. 10.1136/bmj.m1590 32467098 PMC7254147

[B27] Perez-Chada L. M. Salame N. F. Ford A. R. Duffin K. C. Garg A. Gottlieb A. B. (2020). Investigator and patient global assessment measures for psoriasis clinical trials: a systematic review on measurement properties from the international dermatology outcome measures IDEOM initiative. Am. J. Clin. Dermatol 21 (3), 323–338. 10.1007/s40257-019-00496-w 31950353

[B28] Potestio L. Tommasino N. Lauletta G. Feo F. Ruggiero A. Martora F. (2024). Efficacy and safety of deucravacitinib for the management of psoriasis: a drug safety evaluation. Expert Opin. Drug Saf. 23 (6), 677–685. 10.1080/14740338.2024.2351462 38699874

[B29] Qi-Yue Y. Ting Z. Ya-Nan H. Sheng-Jie H. Xuan D. Li H. (2020). From natural dye to herbal medicine: a systematic review of chemical constituents, pharmacological effects and clinical applications of indigo naturalis. Chin. Med. 15 (1), 127. 10.1186/s13020-020-00406-x 33317592 PMC7734464

[B30] Raharja A. Mahil S. K. Barker J. N. (2021). Psoriasis: a brief overview. Clin. Med. (Lond) 21 (3), 170–173. 10.7861/clinmed.2021-0257 34001566 PMC8140694

[B31] Sekhon S. Koo J. (2018). Indirubin: a novel topical agent in the treatment of psoriasis. Br. J. Dermatol 178 (1), 21. 10.1111/bjd.16074 29357584

[B32] Skrek S. Di Lernia V. Beauchet A. Bursztejn A. C. Belloni Fortina A. Lesiak A. (2023). Clinical and epidemiological features of psoriasis exacerbations in children with SARS-CoV-2 infection. J. Eur. Acad. Dermatol Venereol. 37 (10), e1192–e1195. 10.1111/jdv.19261 37326146

[B33] Strober B. van de Kerkhof P. C. M. Callis Duffin K. Poulin Y. Warren R. B. de la Cruz C. (2019). Feasibility and utility of the psoriasis symptom inventory (PSI) in clinical care settings: a study from the International psoriasis council. Am. J. Clin. Dermatol 20 (5), 699–709. 10.1007/s40257-019-00458-2 31228013 PMC6764927

[B34] Su W. Chang Z. E Y. Feng Y. Yao X. Wang M. (2024). Electrospinning and electrospun polysaccharide-based nanofiber membranes: a review. Int. J. Biol. Macromol. 263 (Pt 2), 130335. 10.1016/j.ijbiomac.2024.130335 38403215

[B35] Timis T. Florian I. A. Vesa S. C. Mitrea D. R. Orasan R. I. (2021). An updated guide in the management of psoriasis for every practitioner. Int. J. Clin. Pract. 75 (8), e14290. 10.1111/ijcp.14290 33928703

[B36] Vázquez López F. González-Lara L. Martin J. S. Argenziano G. (2014). Dr K. Holubar (1936-2013). Teaching with dermoscopy: revealing the subsurface morphology of Auspitz's sign and psoriasis. Int. J. Dermatol 53 (5), e322–e324. 10.1111/ijd.12187 24527813

[B37] Wang C. Yang P. Wang J. Liu L. Chen J. Cai X. (2024). Evidence and potential mechanism of action of indigo naturalis and its active components in the treatment of psoriasis. Ann. Med. 56 (1), 2329261. 10.1080/07853890.2024.2329261 39316667 PMC11423532

[B38] Wang P. Gao J. Guo S. Liu H. Cao C. Hong S. (2023). Benefits of topical indigo naturalis nanofibrous patch on psoriatic skin: a transdermal strategy for botanicals. Mater Today Bio 22, 100756. 10.1016/j.mtbio.2023.100756 PMC10430593 37593218

[B39] Xie X. Di T. T. Wang Y. Wang M. X. Meng Y. J. Lin Y. (2018). Indirubin ameliorates imiquimod-induced psoriasis-like skin lesions in mice by inhibiting inflammatory responses mediated by IL-17A-producing γδ T cells. Mol. Immunol. 101, 386–395. 10.1016/j.molimm.2018.07.011 30064075

[B40] Xiong Y. Wang J. Wang S. Li H. Zhou X. (2023). Tryptanthrin ameliorates imiquimod-induced psoriasis in mice by suppressing inflammation and oxidative stress via NF-κB/MAPK/Nrf2 pathways. J. Nat. Med. 77 (1), 188–201. 10.1007/s11418-022-01664-9 36378401

[B41] Xu Y. Lin C. Tan H. Y. Bian Z. X. (2024). The double-edged sword effect of indigo naturalis. Food Chem. Toxicol. 185, 114476. 10.1016/j.fct.2024.114476 38301993

[B42] Xue X. Wu J. Li J. Xu J. Dai H. Tao C. (2018). Indirubin attenuates mouse psoriasis-like skin lesion in a CD274-dependent manner: an achievement of RNA sequencing. Biosci. Rep. 38 (6). 10.1042/BSR20180958 PMC6250808 30341238

[B43] Yu N. Peng C. Zhou J. Gu J. Xu J. Li X. (2023). Measurement properties of the patient global assessment numerical rating scale in moderate-to-severe psoriasis. Br. J. Dermatol 189 (4), 437–446. 10.1093/bjd/ljad188 37310289

[B44] Zhang L. Guo L. Wang L. Jiang X. (2022a). The efficacy and safety of tofacitinib, peficitinib, solcitinib, baricitinib, abrocitinib and deucravacitinib in plaque psoriasis - a network meta-analysis. J. Eur. Acad. Dermatol Venereol. 36 (11), 1937–1946. 10.1111/jdv.18263 35608188

[B45] Zhang Q. Xie J. Li G. Wang F. Lin J. Yang M. (2022b). Psoriasis treatment using indigo naturalis: progress and strategy. J. Ethnopharmacol. 297, 115522. 10.1016/j.jep.2022.115522 35872288

[B46] Zhao J. Xie X. Di T. Liu Y. Qi C. Chen Z. (2021). Indirubin attenuates IL-17A-induced CCL20 expression and production in keratinocytes through repressing TAK1 signaling pathway. Int. Immunopharmacol. 94, 107229. 10.1016/j.intimp.2020.107229 33611057

[B47] Zhou S. Liu Z. Jin Y. Huang Y. Fang Y. Tian H. (2024). Poly (lactic acid) electrospun nanofiber membranes: advanced characterization for biomedical applications with drug loading performance studies. Int. J. Biol. Macromol. 281 (Pt 1), 136188. 10.1016/j.ijbiomac.2024.136188 39368570

